# Supplementary material for: Comparison of clinical characteristics of Zika and dengue symptomatic infections and other acute illnesses of unidentified origin in Mexico
Source: PLoS Negl Trop Dis. 2021 Feb 16;15(2):e0009133. doi: 10.1371/journal.pntd.0009133 (PMC7909682; doi:10.1371/journal.pntd.0009133)
Supplement: S1 Table — (PDF) [file pntd.0009133.s001.pdf]

S1 Table. Description of disability assessment during the first month of follow up using the WHO-DAS tool for disability among 441 patients 12 years and older seeking care within 7 days of onset due to acute episodes of fever and/or rash according to type infection.

Type of Infection		Day 0 (n=406)	Day 7 (n=376)	Day 28 (n=352)
Zika - WHODAS total score	N	37	35	33
	Median	28.330	26.670	23.330
	Min., Max.	(20.00 - 88.33)	(20.00 - 81.67)	(20.00 - 70.00)
Dengue - WHODAS total score	N	73	56	54
	Median	40.000	34.165	27.500
	Min., Max.	(20.00 - 85.00)	(20.00 - 73.33)	(20.00 - 73.33)
AIUO - WHODAS total score	N	296	285	265
	Median	33.330	26.670	23.330
	Min., Max.	(20.00 - 91.67)	(20.00 - 95.00)	(20.00 - 80.00)
Wilcoxons test p-values ZIKA vs DENGUE[1]		**0.0171**	1.0000	1.0000
		(0.0002)	(0.1462)	(0.5335)
Wilcoxons test p-values ZIKA vs AIUO[1]		1.0000	1.0000	1.0000
		(0.0221)	(0.8256)	(0.7135)
Wilcoxons test p-values DENGUE vs AIUO[1]		0.0766	1.0000	1.0000
		(0.0011)	(0.0154)	(0.0909)

[1]P-values are presented as adjusted (unadjusted).
