# Supplementary material for: Comparison of clinical characteristics of Zika and dengue symptomatic infections and other acute illnesses of unidentified origin in Mexico
Source: PLoS Negl Trop Dis. 2021 Feb 16;15(2):e0009133. doi: 10.1371/journal.pntd.0009133 (PMC7909682; doi:10.1371/journal.pntd.0009133)
Supplement: S2 Table — (PDF) [file pntd.0009133.s002.pdf]

S2 Table. Clinical laboratory blood tests at baseline visit of patients 12 years and older seeking care within 7 days of onset due to acute episodes of fever and/or rash (N=406).

**Laboratory parameter**[1]	**Zika (n=37)**	**Dengue (n=73)**[2]	**Acute Illnesses of Unidentified Origin (n=296)**[3]	**p-value**[7] ZIKA vs DENGUE	**p-value**[7] ZIKA vs AIUO	**p-value**[7] DENGUE vs AIUO
Hemoglobin (mg/dL)	13.4 (12.8-14.5)	14.20 (13.2-15.35)	13.75 (12.9-14.8)	1.0000 (0.0733)	1.0000 (0.6416)	1.0000 (0.0455)
Hematocrit (%)	40.6 (38.2-42.5)	42.1 (39.25-44.9)	41 (38.7-44.3)	1.0000 (0.1014)	1.0000 (0.2679)	1.0000 (0.2749)
Platelets (10^3 cell/μl)	244 (219-284)	98 (40-125.5)	234 (185-288)	**<0.0001** (<0.0001)	1.0000 (0.2184)	**<0.0001** (<0.0001)
Leukocytes (10^3 cell/μl)	5.4 (4.3-6.5)	3.7 (2.75-5.1)	7.2 (5.4-9.5)	**0.0016** (<0.0001)	**0.0452** (0.0006)	**<0.0001** (<0.0001)
Neutrophils (%)	58 (50-64)	34 (27-51)	62 (51-69)	**0.0002** (<0.0001)	1.0000 (0.2514)	**<0.0001** (<0.0001)
Lymphocytes (%)	29 (20-35)	41 (19.5-50)	25 (18-34)	0.3756 (0.0066)	1.0000 (0.2709)	**0.0014** (<0.0001)
ALT (IU/L)	39 (28-54)	76 (44-125)	42 (27-68)[4]	**0.0002** (<0.0001)	1.0000 (0.5757)	**<0.0001** (<0.0001)
Creatinine Kinase (IU/L)	68 (57-120)	78 (58-142)	75 (53.75-104.5)[5]	1.0000 (0.3426)	1.0000 (0.9051)	1.0000 (0.2139)
C-Reactive Protein (mg/L)	0.9 (0.3-2.3)	1.2 (0.6-2.4)	2.1 (0.55-5.6)[6]	1.0000 (0.1040)	0.3550 (0.0061)	0.9875 (0.0186)
Erythrocyte Sedimentation Rate (mm/hr)	22 (11-37)	14 (10-21)[2]	23 (15-36.75)[3]	1.0000 (0.0333)	1.0000 (0.5055)	**<0.0001** (<0.0001)

[1]All parameters summarized using medians and 25th and 75th percentiles. [2]Complete blood counts and Erythrocyte Sedimentation Rate missing values in two participants. [3]Complete blood counts and Erythrocyte Sedimentation Rate missing values in six participants. [4]Three ALT missing values (n=293). [5] Four CPK missing values. [6]One missing value in C-reactive protein. [7]P-values are presented as adjusted (unadjusted).
