# Supplementary material for: Comparison of clinical characteristics of Zika and dengue symptomatic infections and other acute illnesses of unidentified origin in Mexico
Source: PLoS Negl Trop Dis. 2021 Feb 16;15(2):e0009133. doi: 10.1371/journal.pntd.0009133 (PMC7909682; doi:10.1371/journal.pntd.0009133)
Supplement: S3 Table — (PDF) [file pntd.0009133.s003.pdf]

S3 Table. Distribution and characteristics of self-reported signs and symptoms at day 3 after baseline visit of patients 12 years and older seeking care within 7 days of onset due to acute episodes of fever and/or (N=383).

	**Confirmed Zika Infection (n=36)**	**Confirmed Dengue Infection (n=62)**	**Acute Illnesses of Unidentified Origin (n=285)**	**p-value[2]** ZIKA vs DENGUE	**p-value[2]** ZIKA vs AIUO	**p-value[2]** DENGUE vs AIUO
Rash (self-reported)[1]	20 (55.6%)	35 (56.5%)	77 (27.0%)	1.0000 (1.0000)	0.0851 (0.0009)	**0.0019** (<0.0001)
Arthralgia[1]	16 (44.4%)	25 (40.3%)	136 (47.7%)	1.0000 (0.8320)	1.0000 (0.7272)	1.0000 (0.3266)
Myalgia[1]	22 (61.1%)	30 (48.4%)	191 (67.0%)	1.0000 (0.2943)	1.0000 (0.5747)	0.7592 (0.0083)
Conjunctivitis[1]	16 (44.4%)	6 (9.7%)	63 (22.1%)	**0.0124** (0.0001)	0.6105 (0.0066)	1.0000 (0.0338)
Headache[1]	18 (50.0%)	29 (46.8%)	195 (68.4%)	1.0000 (0.8350)	1.0000 (0.0383)	0.1872 (0.0019)
Malaise[1]	28 (77.8%)	39 (62.9%)	204 (71.6%)	1.0000 (0.1766)	1.0000 (0.5543)	1.0000 (0.2206)
Muscular weakness	9 (25.0%)	20 (32.3%)	98 (34.4%)	1.0000 (0.4984)	1.0000 (0.3482)	1.0000 (0.8825)
Fatigue	18 (50.0%)	37 (59.7%)	165 (57.9%)	1.0000 (0.4019)	1.0000 (0.3776)	1.0000 (0.8872)
Back pain	21 (58.3%)	32 (51.6%)	180 (63.2%)	1.0000 (0.5368)	1.0000 (0.5872)	1.0000 (0.1135)
Peri-orbital pain	13 (36.1%)	19 (30.6%)	133 (46.7%)	1.0000 (0.6568)	1.0000 (0.2871)	1.0000 (0.0238)
Altered Behavior or Personality	5 (13.9%)	10 (16.1%)	64 (22.5%)	1.0000 (1.0000)	1.0000 (0.2871)	1.0000 (0.3085)
Confusion/Disorientation	3 (8.3%)	7 (11.3%)	72 (25.3%)	1.0000 (0.7414)	1.0000 (0.0217)	1.0000 (0.0187)
Stiff neck	4 (11.1%)	12 (19.4%)	51 (17.9%)	1.0000 (0.3982)	1.0000 (0.4799)	1.0000 (0.8558)

	**Confirmed Zika Infection (n=36)**	**Confirmed Dengue Infection (n=62)**	**Acute Illnesses of Unidentified Origin (n=285)**	**p-value[2]** ZIKA vs DENGUE	**p-value[2]** ZIKA vs AIUO	**p-value[2]** DENGUE vs AIUO
Sore throat	13 (36.1%)	18 (29.0%)	140 (49.1%)	1.0000 (0.5046)	1.0000 (0.1590)	0.4415 (0.0047)
Mouth ulcers	3 (8.3%)	8 (12.9%)	44 (15.4%)	1.0000 (0.7414)	1.0000 (0.3242)	1.0000 (0.6983)
Nausea	11 (30.6%)	14 (22.6%)	102 (35.8%)	1.0000 (0.4721)	1.0000 (0.5836)	1.0000 (0.0533)
Vomiting	2 (5.6%)	6 (9.7%)	25 (8.8%)	1.0000 (0.7064)	1.0000 (0.7520)	1.0000 (0.8071)
Diarrhea	2 (5.6%)	18 (29.0%)	55 (19.3%)	0.7363 (0.0080)	1.0000 (0.0392)	1.0000 (0.1204)
Itchiness	18 (50.0%)	33 (53.2%)	94 (33.0%)	1.0000 (0.8350)	1.0000 (0.0621)	0.3390 (0.0035)
Cough	10 (27.8%)	21 (33.9%)	141 (49.5%)	1.0000 (0.6535)	1.0000 (0.0202)	1.0000 (0.0346)
Bleeding	2 (5.6%)	3 (4.8%)	15 (5.3%)	1.0000 (1.0000)	1.0000 (1.0000)	1.0000 (1.0000)
Petechiae (self-reported)	1 (2.8%)	14 (22.6%)	9 (3.2%)	0.7592 (0.0084)	1.0000 (1.0000)	**0.0002** (<0.0001)
Photophobia	22 (61.1%)	23 (37.1%)	138 (48.4%)	1.0000 (0.0348)	1.0000 (0.1614)	1.0000 (0.1226)
Difficulty Walking	10 (27.8%)	20 (32.3%)	101 (35.4%)	1.0000 (0.8205)	1.0000 (0.4578)	1.0000 (0.6624)
Difficult Standing Upright/Hunched	12 (33.3%)	31 (50.0%)	115 (40.4%)	1.0000 (0.1405)	1.0000 (0.4727)	1.0000 (0.2012)

Note: Patients that have multiple diagnoses have been removed.

[1]One or more of these were part of entry criteria. [2]P-values are presented as adjusted (unadjusted).
