# Supplementary material for: Comparison of clinical characteristics of Zika and dengue symptomatic infections and other acute illnesses of unidentified origin in Mexico
Source: PLoS Negl Trop Dis. 2021 Feb 16;15(2):e0009133. doi: 10.1371/journal.pntd.0009133 (PMC7909682; doi:10.1371/journal.pntd.0009133)
Supplement: S4 Table — (PDF) [file pntd.0009133.s004.pdf]

S4 Table. Distribution and characteristics of physical exam 3 days after the first visit of patients 12 years and older seeking care within 7 days of onset due to acute episodes of fever and/or rash (N=383).

	**Confirmed Zika Infection (n=36)**	**Confirmed Dengue Infection (n=62)**	**Acute Illnesses of Unidentified Origin (n=285)**	**p-value[1]** ZIKA vs DENGUE	**p-value[1]** ZIKA vs AIUO	**p-value[1]** DENGUE vs AIUO
Rash at physical exam	17 (47.2%)	36 (58.1%)	74 (26.0%)	1.0000 (0.4006)	0.9433 (0.0106)	**0.0002** (<0.0001)
Maculopapular	8 (47.1%)	4 (11.1%)	19 (25.7%)	0.9467 (0.0108)	1.0000 (0.1382)	1.0000 (0.0869)
Petechial	0 (0.0%)	8 (22.2%)	2 (2.7%)	1.0000 (0.0438)	1.0000 (1.0000)	0.1859 (0.0019)
Erythematous	9 (52.9%)	29 (80.6%)	53 (71.6%)	1.0000 (0.0526)	1.0000 (0.1564)	1.0000 (0.3590)
Other - combined with Bruising	1 (5.9%)	1 (2.8%)	2 (2.7%)	1.0000 (0.5428)	1.0000 (0.4664)	1.0000 (1.0000)
Injected conjuntivae	8 (22.2%)	3 (4.8%)	56 (19.6%)	1.0000 (0.0165)	1.0000 (0.6639)	0.4194 (0.0044)
Uveitis	1 (2.8%)	0 (0.0%)	20 (7.0%)	1.0000 (0.3673)	1.0000 (0.4885)	1.0000 (0.0314)
Petechiae at physical exam	0 (0.0%)	15 (24.2%)	8 (2.8%)	0.0794 (0.0008)	1.0000 (0.6045)	**0.00002** (<0.0001)
Lymphadenopathy	12 (33.3%)	28 (45.2%)	117 (41.1%)	1.0000 (0.2910)	1.0000 (0.4712)	1.0000 (0.5721)
Any neurological abnormal physical finding	1 (2.8%)	6 (9.7%)	12 (4.2%)	1.0000 (0.2564)	1.0000 (1.0000)	1.0000 (0.1070)

[1]P-values are presented as adjusted (unadjusted).
