# Supplementary material for: Comparison of clinical characteristics of Zika and dengue symptomatic infections and other acute illnesses of unidentified origin in Mexico
Source: PLoS Negl Trop Dis. 2021 Feb 16;15(2):e0009133. doi: 10.1371/journal.pntd.0009133 (PMC7909682; doi:10.1371/journal.pntd.0009133)
Supplement: S5 Table — (PDF) [file pntd.0009133.s005.pdf]

S5 Table. Distribution and characteristics of self-reported signs and symptoms at day 7 after baseline visit of patients 12 years and older seeking care within 7 days of onset due to acute episodes of fever and/or rash (N=376).

	**Confirmed Zika Infection (n=35)**	**Confirmed Dengue Infection (n=56)**	**Acute Illnesses of Unidentified Origin (n=285)**	**p-value**[2] ZIKA vs DENGUE	**p-value**[2] ZIKA vs AIUO	**p-value**[2] DENGUE vs AIUO
Rash (self-reported)[1]	10 (28.6%)	17 (30.4%)	39 (13.7%)	1.0000 (1.0000)	1.0000 (0.0423)	0.5167 (0.0048)
Arthralgia[1]	8 (22.9%)	18 (32.1%)	107 (37.5%)	1.0000 (0.4748)	1.0000 (0.0958)	1.0000 (0.5443)
Myalgia[1]	16 (45.7%)	26 (46.4%)	146 (51.2%)	1.0000 (1.0000)	1.0000 (0.5933)	1.0000 (0.5601)
Conjunctivitis[1]	7 (20.0%)	5 (8.9%)	51 (17.9%)	1.0000 (0.2014)	1.0000 (0.8160)	1.0000 (0.1155)
Headache[1]	14 (40.0%)	26 (46.4%)	163 (57.2%)	1.0000 (0.6650)	1.0000 (0.0708)	1.0000 (0.1445)
Malaise[1]	12 (34.3%)	34 (60.7%)	139 (48.8%)	1.0000 (0.0182)	1.0000 (0.1107)	1.0000 (0.1098)
Muscular weakness	4 (11.4%)	19 (33.9%)	87 (30.5%)	1.0000 (0.0244)	1.0000 (0.0171)	1.0000 (0.6372)
Fatigue	13 (37.1%)	34 (60.7%)	130 (45.6%)	1.0000 (0.0333)	1.0000 (0.3726)	1.0000 (0.0416)
Back pain	13 (37.1%)	23 (41.1%)	147 (51.6%)	1.0000 (0.8264)	1.0000 (0.1511)	1.0000 (0.1881)
Peri-orbital pain	8 (22.9%)	13 (23.2%)	94 (33.0%)	1.0000 (1.0000)	1.0000 (0.2543)	1.0000 (0.1601)
Altered Behavior or Personality	3 (8.6%)	4 (7.1%)	45 (15.8%)	1.0000 (1.0000)	1.0000 (0.3241)	1.0000 (0.0993)
Confusion/Disorientation	4 (11.4%)	10 (17.9%)	64 (22.5%)	1.0000 (0.5539)	1.0000 (0.1875)	1.0000 (0.5947)
Stiff neck	5 (14.3%)	3 (5.4%)	36 (12.6%)	1.0000 (0.2522)	1.0000 (0.7888)	1.0000 (0.1663)

	Confirmed Zika Infection (n=35)	Confirmed Dengue Infection (n=56)	Acute Illnesses of Unidentified Origin (n=285)	**p-value[2]** ZIKA vs DENGUE	**p-value[2]** ZIKA vs AIUO	**p-value[2]** DENGUE vs AIUO
Sore throat	8 (22.9%)	11 (19.6%)	96 (33.7%)	1.0000 (0.7931)	1.0000 (0.2519)	1.0000 (0.0411)
Mouth ulcers	1 (2.9%)	2 (3.6%)	30 (10.5%)	1.0000 (1.0000)	1.0000 (0.2249)	1.0000 (0.1328)
Nausea	11 (31.4%)	16 (28.6%)	71 (24.9%)	1.0000 (0.8162)	1.0000 (0.4152)	1.0000 (0.6154)
Vomiting	3 (8.6%)	3 (5.4%)	13 (4.6%)	1.0000 (0.6721)	1.0000 (0.3985)	1.0000 (0.7335)
Diarrhea	4 (11.4%)	8 (14.3%)	39 (13.7%)	1.0000 (0.7614)	1.0000 (1.0000)	1.0000 (0.8352)
Itchiness	14 (40.0%)	23 (41.1%)	79 (27.7%)	1.0000 (1.0000)	1.0000 (0.1662)	1.0000 (0.0554)
Cough	10 (28.6%)	18 (32.1%)	127 (44.6%)	1.0000 (0.8172)	1.0000 (0.1022)	1.0000 (0.1038)
Bleeding	0 (0.0%)	5 (8.9%)	16 (5.6%)	1.0000 (0.1522)	1.0000 (0.2334)	1.0000 (0.3607)
Petechiae (self-reported)	1 (2.9%)	3 (5.4%)	6 (2.1%)	1.0000 (1.0000)	1.0000 (0.5591)	1.0000 (0.1702)
Photophobia	12 (34.3%)	16 (28.6%)	111 (38.9%)	1.0000 (0.6429)	1.0000 (0.7134)	1.0000 (0.1737)
Difficulty Walking	7 (20.0%)	20 (35.7%)	86 (30.2%)	1.0000 (0.1570)	1.0000 (0.2419)	1.0000 (0.4319)
Difficult Standing Upright/Hunched	8 (22.9%)	25 (44.6%)	93 (32.6%)	1.0000 (0.0447)	1.0000 (0.3350)	1.0000 (0.0923)

Note: Patients that have multiple diagnoses have been removed.

[1]One or more of these were part of entry criteria. [2]P-values are presented as adjusted (unadjusted).
