# Supplementary material for: Comparison of clinical characteristics of Zika and dengue symptomatic infections and other acute illnesses of unidentified origin in Mexico
Source: PLoS Negl Trop Dis. 2021 Feb 16;15(2):e0009133. doi: 10.1371/journal.pntd.0009133 (PMC7909682; doi:10.1371/journal.pntd.0009133)
Supplement: S6 Table — (PDF) [file pntd.0009133.s006.pdf]

**S6 Table. Distribution and characteristics of physical exam 7 days after the first visit of patients 12 years and older seeking care within 7 days of onset due to acute episodes of fever and/or rash (N=376).**

| | **Confirmed Zika Infection (n=35)** | **Confirmed Dengue Infection (n=56)** | **Acute Illnesses of Unidentified Origin (n=285)** | **p-value[1]** ZIKA vs DENGUE | **p-value[1]** ZIKA vs AIUO | **p-value[1]** DENGUE vs AIUO |
|---|---|---|---|---|---|---|
| Rash at physical exam | 7 (20.0%) | 17 (30.4%) | 36 (12.6%) | 1.0000 (0.3335) | 1.0000 (0.2891) | 0.2140 (0.0020) |
| Maculopapular | 5 (71.4%) | 3 (17.6%) | 8 (22.2%) | 1.0000 (0.0207) | 1.0000 (0.0190) | 1.0000 (1.0000) |
| Petechial | 1 (14.3%) | 1 (5.9%) | 2 (5.6%) | 1.0000 (0.5072) | 1.0000 (0.4214) | 1.0000 (1.0000) |
| Erythematous | 2 (28.6%) | 14 (82.4%) | 26 (72.2%) | 1.0000 (0.0207) | 1.0000 (0.0398) | 1.0000 (0.5111) |
| Other - combined with Bruising | 0 (0.0%) | 0 (0.0%) | 1 (2.8%) | 1.0000 (1.0000) | 1.0000 (1.0000) | 1.0000 (1.0000) |
| Injected conjuntivae | 6 (17.1%) | 4 (7.1%) | 37 (13.0%) | 1.0000 (0.1748) | 1.0000 (0.4416) | 1.0000 (0.2670) |
| Uveitis | 1 (2.9%) | 0 (0.0%) | 8 (2.8%) | 1.0000 (0.3846) | 1.0000 (1.0000) | 1.0000 (0.3622) |
| Petechiae at physical exam | 1 (2.9%) | 1 (1.8%) | 7 (2.5%) | 1.0000 (1.0000) | 1.0000 (1.0000) | 1.0000 (1.0000) |
| Lymphadenopathy | 10 (28.6%) | 13 (23.2%) | 92 (32.3%) | 1.0000 (0.6242) | 1.0000 (0.7057) | 1.0000 (0.2070) |
| Any neurological abnormal physical finding | 1 (2.9%) | 7 (12.5%) | 41 (14.4%) | 1.0000 (0.1463) | 1.0000 (0.0629) | 1.0000 (0.8352) |

[1]P-values are presented as adjusted (unadjusted).
