# Supplementary material for: Comparison of clinical characteristics of Zika and dengue symptomatic infections and other acute illnesses of unidentified origin in Mexico
Source: PLoS Negl Trop Dis. 2021 Feb 16;15(2):e0009133. doi: 10.1371/journal.pntd.0009133 (PMC7909682; doi:10.1371/journal.pntd.0009133)
Supplement: S7 Table — (PDF) [file pntd.0009133.s007.pdf]

S7 Table. Distribution and characteristics of self-reported signs and symptoms at day 28 after baseline visit of patients 12 years and older seeking care within 7 days of onset due to acute episodes of fever and/or rash (N=352).

	**Confirmed Zika Infection (n=33)**	**Confirmed Dengue Infection (n=54)**	**Acute Illnesses of Unidentified Origin (n=265)**	**p-value[2]** ZIKA vs DENGUE	**p-value[2]** ZIKA vs AIUO	**p-value[2]** DENGUE vs AIUO
Rash (self-reported)[1]	3 (9.1%)	3 (5.6%)	23 (8.7%)	1.0000 (0.6693)	1.0000 (1.0000)	1.0000 (0.5904)
Arthralgia[1]	12 (36.4%)	14 (25.9%)	76 (28.7%)	1.0000 (0.3404)	1.0000 0.4186	1.0000 (0.7424)
Myalgia[1]	15 (45.5%)	13 (24.1%)	102 (38.5%)	1.0000 (0.0578)	1.0000 (0.4547)	1.0000 (0.0612)
Conjunctivitis[1]	4 (12.1%)	4 (7.4%)	28 (10.6%)	1.0000 (0.4713)	1.0000 (0.7662)	1.0000 (0.6227)
Headache[1]	11 (33.3%)	18 (33.3%)	119 (44.9%)	1.0000 (1.0000)	1.0000 (0.2645)	1.0000 (0.1328)
Malaise[1]	9 (27.3%)	15 (27.8%)	91 (34.3%)	1.0000 (1.0000)	1.0000 (0.5579)	1.0000 (0.4286)
Muscular weakness	4 (12.1%)	9 (16.7%)	55 (20.8%)	1.0000 (0.7587)	1.0000 (0.3533)	1.0000 (0.5789)
Fatigue	12 (36.4%)	21 (38.9%)	95 (35.8%)	1.0000 (1.0000)	1.0000 (1.0000)	1.0000 (0.7565)
Back pain	13 (39.4%)	17 (31.5%)	127 (47.9%)	1.0000 (0.4916)	1.0000 (0.4599)	1.0000 (0.0351)
Peri-orbital pain	8 (24.2%)	7 (13.0%)	79 (29.8%)	1.0000 (0.2428)	1.0000 (0.6849)	1.0000 (0.0112)
Altered Behavior or Personality	4 (12.1%)	7 (13.0%)	35 (13.2%)	1.0000 (1.0000)	1.0000 (1.0000)	1.0000 (1.0000)
Confusion/Disorientation	2 (6.1%)	4 (7.4%)	43 (16.2%)	1.0000 (1.0000)	1.0000 (0.1940)	1.0000 (0.1381)
Stiff neck	4 (12.1%)	4 (7.4%)	35 (13.2%)	1.0000 (0.4713)	1.0000 (1.0000)	1.0000 (0.3603)

	**Confirmed Zika Infection (n=33)**	**Confirmed Dengue Infection (n=54)**	**Acute Illnesses of Unidentified Origin (n=265)**	**p-value[2]** ZIKA vs DENGUE	**p-value[2]** ZIKA vs AIUO	**p-value[2]** DENGUE vs AIUO
Sore throat	10 (30.3%)	11 (20.4%)	84 (31.7%)	1.0000 (0.3130)	1.0000 (1.0000)	1.0000 (0.1050)
Mouth ulcers	3 (9.1%)	2 (3.7%)	16 (6.0%)	1.0000 (0.3631)	1.0000 (0.4525)	1.0000 (0.7478)
Nausea	5 (15.2%)	6 (11.1%)	45 (17.0%)	1.0000 (0.7412)	1.0000 (1.0000)	1.0000 (0.4142)
Vomiting	0 (0.0%)	1 (1.9%)	16 (6.0%)	1.0000 (1.0000)	1.0000 0.2320	1.0000 (0.3239)
Diarrhea	6 (18.2%)	6 (11.1%)	33 (12.5%)	1.0000 (0.3594)	1.0000 (0.4085)	1.0000 (1.0000)
Itchiness	4 (12.1%)	10 (18.5%)	60 (22.6%)	1.0000 (0.5536)	1.0000 (0.2590)	1.0000 (0.5907)
Cough	5 (15.2%)	8 (14.8%)	95 (35.8%)	1.0000 (1.0000)	1.0000 (0.0185)	0.3045 (0.0023)
Bleeding	2 (6.1%)	1 (1.9%)	11 (4.2%)	1.0000 (0.5545)	1.0000 (0.6434)	1.0000 (0.6984)
Petechiae (self-reported)	0 (0.0%)	0 (0.0%)	6 (2.3%)	1.0000 (1.0000)	1.0000 (1.0000)	1.0000 (0.5944)
Photophobia	9 (27.3%)	11 (20.4%)	84 (31.7%)	1.0000 (0.6002)	1.0000 (0.6932)	1.0000 (0.1050)
Difficulty Walking	5 (15.2%)	9 (16.7%)	56 (21.1%)	1.0000 (1.0000)	1.0000 (0.5003)	1.0000 (0.5788)
Difficult Standing Upright/Hunched	7 (21.2%)	12 (22.2%)	63 (23.8%)	1.0000 (1.0000)	1.0000 (0.8308)	1.0000 (0.8622)

Note: Patients that have multiple diagnoses have been removed.

[1]One or more of these were part of entry criteria. [2]P-values are presented as adjusted (unadjusted).
