# Supplementary material for: Comparison of clinical characteristics of Zika and dengue symptomatic infections and other acute illnesses of unidentified origin in Mexico
Source: PLoS Negl Trop Dis. 2021 Feb 16;15(2):e0009133. doi: 10.1371/journal.pntd.0009133 (PMC7909682; doi:10.1371/journal.pntd.0009133)
Supplement: S8 Table — (PDF) [file pntd.0009133.s008.pdf]

S8 Table. Distribution and characteristics of physical exam 28 days after the first visit of patients 12 years and older seeking care within 7 days of onset due to acute episodes of fever and/or rash (N=352).

	**Confirmed Zika Infection (n=33)**	**Confirmed Dengue Infection (n=54)**	**Acute Illnesses of Unidentified Origin (n=265)**	**p-value[1]** ZIKA vs DENGUE	**p-value[1]** ZIKA vs AIUO	**p-value[1]** DENGUE vs AIUO
Rash at physical exam	2 (6.1%)	2 (3.7%)	12 (4.5%)	1.0000 (0.6323)	1.0000 (0.6591)	1.0000 (1.0000)
Maculopapular	1 (50.0%)	1 (50.0%)	3 (25.0%)	1.0000 (1.0000)	1.0000 (0.5055)	1.0000 (0.5055)
Petechial	0 (0.0%)	0 (0.0%)	0 (0.0%)	1.0000 (1.0000)	1.0000 (1.0000)	1.0000 (1.0000)
Erythematous	1 (50.0%)	1 (50.0%)	8 (66.7%)	1.0000 (1.0000)	1.0000 (1.0000)	1.0000 (1.0000)
Other - combined with Bruising	0 (0.0%)	0 (0.0%)	2 (16.7%)	1.0000 (1.0000)	1.0000 (1.0000)	1.0000 (1.0000)
Injected conjuntivae	2 (6.1%)	4 (7.4%)	13 (4.9%)	1.0000 (1.0000)	1.0000 (0.6759)	1.0000 (0.5030)
Uveitis	0 (0.0%)	0 (0.0%)	3 (1.1%)	1.0000 (1.0000)	1.0000 (1.0000)	1.0000 (1.0000)
Petechiae at physical exam	0 (0.0%)	0 (0.0%)	3 (1.1%)	1.0000 (1.0000)	1.0000 (1.0000)	1.0000 (1.0000)
Lymphadenopathy	4 (12.1%)	6 (11.1%)	51 (19.2%)	1.0000 (1.0000)	1.0000 (0.4746)	1.0000 (0.1768)
Any neurological abnormal physical finding	2 (6.1%)	2 (3.7%)	26 (9.8%)	1.0000 (0.6323)	1.0000 (0.7519)	1.0000 (0.1913)

[1]P-values are presented as adjusted (unadjusted).
