# Supplementary material for: Comparison of clinical characteristics of Zika and dengue symptomatic infections and other acute illnesses of unidentified origin in Mexico
Source: PLoS Negl Trop Dis. 2021 Feb 16;15(2):e0009133. doi: 10.1371/journal.pntd.0009133 (PMC7909682; doi:10.1371/journal.pntd.0009133)
Supplement: S9 Table — (PDF) [file pntd.0009133.s009.pdf]

S9 Table. Clinical laboratory blood tests at Day 28 visit of patients 12 years and older seeking care within 7 days of onset due to acute episodes of fever and/or rash (N=352)

**Laboratory parameter**[1]	**Zika (n=33)**	**Dengue (n=54)**	**Acute Illnesses of Unidentified Origin (n=265)**[2]	**p-value**[6] ZIKA vs DENGUE	**p-value**[6] ZIKA vs AIUO	**p-value**[6] DENGUE vs AIUO
Hemoglobin (mg/dL)	13.4 (13-14.3)	13.55 (12.8-14.675)	13.4 (12.6-14.5)	1.0000 (0.7727)	1.0000 (0.6112)	1.0000 (0.3363)
Hematocrit (%)	40.1 (38.3-43.7)	40.4 (37.975-43.2)	39.9 (38.1-42.925)	1.0000 (0.6523)	1.0000 (0.9956)	1.0000 (0.5471)
Platelets (10^3 cell/µl)	265 (226-332)	219.5 (194.25-253.75)	253.5 (209.25-301)	**0.0129** (<0.0001)	1.0000 (0.0782)	0.0963 (0.0007)
Leukocytes (10^3 cell/µl)	6.3 (5.8-7.3)	7.6 (6.1-8.35)	6.95 (5.8-8.4)	1.0000 (0.0887)	1.0000 (0.2598)	1.0000 (0.4426)
Neutrophils (%)	54 (51-60)	58 (50.25-65)	59 (53-65)	1.0000 (0.2027)	1.0000 (0.0079)	1.0000 (0.3530)
Lymphocytes (%)	36 (30-40)	30 (36-35.75)	30 (25-36)	1.0000 (0.0196)	0.1686 (0.0012)	1.0000 (0.5701)
ALT (IU/L)	31 (25-47)	36.5 (28-55.5)	32 (26-49)[3]	1.0000 (0.2060)	1.0000 (0.8081)	1.0000 (0.1219)
Creatinine Kinase (IU/L)	99 (62-132)	72 (51.25-92)	77.5 (54-115)[4]	1.0000 (0.0159)	1.0000 (0.1046)	1.0000 (0.1554)
C-Reactive Protein (mg/L)	0.2 (0.2-0.5)	0.5 (0.5-0.8)[5]	0.5 (0.2-0.8)[4]	1.0000 (0.0083)	1.0000 (0.0449)	1.0000 (0.2261)
Erythrocyte Sedimentation Rate (mm/hr)	21 (8-31)	19 (11.5-30)	18 (11-28)[2]	1.0000 (0.7066)	1.0000 (0.8494)	1.0000 (0.8990)

[1]All parameters summarized using medians and 25th and 75th percentiles. [2]Complete blood counts and Erythrocyte Sedimentation Rate missing values in five participants. [3]Two ALT missing values (n=263). [4]Three missing values in CPK and C-reactive protein. [5]One missing value in C-reactive protein. [6]P-values are presented as adjusted (unadjusted).
