# Supplementary material for: Comparison of clinical characteristics of Zika and dengue symptomatic infections and other acute illnesses of unidentified origin in Mexico
Source: PLoS Negl Trop Dis. 2021 Feb 16;15(2):e0009133. doi: 10.1371/journal.pntd.0009133 (PMC7909682; doi:10.1371/journal.pntd.0009133)
Supplement: Attachment — Submitted filename: Zik01_Plos_Main Symptoms_reviewers response _210108.docx [file pntd.0009133.s010.docx]

January 8^th^, 2020

Shaden Kamhawi

Paul Brindley

Peter J. Hotez

Editors-in-Chief

David Joseph Diemert, M.D.

Associate Editor

Amy Morrison

Deputy Editor

PLOS Neglected Tropical Diseases

Dear editors,

We appreciate the opportunity to resubmit our manuscript titled “Comparison of clinical characteristics of Zika and dengue symptomatic infections and other acute illnesses of unidentified origin in Mexico”. We are grateful to the reviewers for their thoughtful comments, as these helped to improve our work.

On behalf of my co-authors, I am resubmitting a corrected version, please find responses in the following pages to each comment from reviewers and a description of the corresponding changes made in the manuscript.

Sincerely,

Dr. Pablo F Belaunzarán-Zamudio

**Reviewers' comments:**

Reviewer #1: -Background: remove "comma" after clinical description

***Author’s response:*** *Deleted*

-Punctuation marks need to be reviewed: missing commas or passive voice sentences

***Author’s response:*** *We carefully reviewed the manuscript for English quality, readability and comprehensibility. The native English-speakers co-authors in the manuscript put attention to any potential issues that might have skipped their attention in the previous version. The reviewers will notice in track changes on different sections of the manuscript these modifications.*

Reviewer #2: The results are appropriate. Some repetition could be reduced, and the supplemental tables need to be properly labeled with footnotes defined.

***Author´s response:*** *We reviewed and re-ordered some sections to avoid repetition. According to the guideline we listed supporting information captions at the end of the manuscript file, however verifying the published articles and following reviewer recommendation, please find the updated version of the supporting information including captions.*

Reviewer #2: The paper would benefit from editing by a native English speaker.

***Author’s response:*** *We carefully reviewed the manuscript for English quality and comprehensibility. The native English-speakers co-authors have considered any potential issues that might have skipped their attention in the previous version. The reviewers will notice in track changes on different sections of the manuscript these modifications.*

Reviewer #2: In the abstract, laboratory diagnosis needs to be described.

***Author’s response:*** *We added in the abstract section “We identified Zika, dengue chikungunya by RT-PCR in serum and urine*”

It is not clear why 8.6% of confirmed dengue and zika cases were excluded, please clarify.

***Author’s response:*** *Thank you for this comment. We corrected this information from the abstract section. The correct information is described in the Results section, stating that we could not classify 34 (7.7%) events due to missing data, hence we excluded them.*

Other AFI are not included in the abstract and could be removed from the methods.

***Author’s response:*** *The sentence “Most episodes (67%) consistent with the PAHO definition of probable Zika could not be confirmed as due to any flavivirus” refers to the acute illnesses of unidentified origin (AIUO) group. We modified this sentence in the abstract and added the acronym (AIUO).*

Clarify when the follow-up visits occurred. The abstract says 3, 7, and 28 days and the methods up to 6 months.

***Author’s response****: We followed the complete cohort for 180 days (6 months). In this manuscript we show the clinical characterization of day 3,7, and 28, because no signs or symptoms directly attributable to the disease persisted up to day 180. We modified the Abstract section accordingly and added this clarification in the Results section.*

Remove the 1 chikungunya case form table 1. Not much can be said from an n=1 and it is distracting.

***Author’s response***: Deleted

A brief description of what the disability assessment measures would be helpful.

***Author’s response***: *We assessed the impact of the disease using the World Health Organization Disability Assessment Schedule 2.0 (WHODAS 2).* *We added a brief explanation of the WHODAS 2 instrument in the procedure section: “The WHODAS 2 is an instrument designed to provide a cross-cultural standardized method for measuring activity limitations and participation restrictions irrespective of the individual´s medical diagnosis [15]. The instrument evaluates 6 domains of functioning: cognition, mobility, self-care, interaction with other people, life activities, and participation; and provides a score to produce a standardized and comparable disability measure”*

The differences in the frequency of rash are repeated in the results in two different places, I suggest you combined and discuss it in the same paragraph, otherwise it seems repetitive.

***Author’s response***: *We re-ordered and re-grouped some paragraphs in the Results section to make clear that we are describing two different measurements of type of rash: the first was self-reported and the second at physical exam. We hope these changes improve readability and comprehension.*

The supplemental tables need titles.

***Author’s response***: As mentioned above, a*ccording to the guideline we listed supporting information captions at the end of the manuscript file, however verifying the published articles and following reviewer recommendation, please find the updated version of the supporting information including captions.*
